# Supplementary material for: Evidence of reactivation of a hydrothermal system from seismic anisotropy changes
Source: Nat Commun. 2019 Nov 21;10:5278. doi: 10.1038/s41467-019-13156-8 (PMC6872732; doi:10.1038/s41467-019-13156-8)
Supplement: Supplementary file 1 — Supplementary Information [file 41467_2019_13156_MOESM1_ESM.pdf]

nature

Supplementary Information for: Evidence of reactivation of a hydrothermal system from seismic anisotropy changes

Maria Saade,[1] Kohtaro Araragi,[2] Jean Paul Montagner,[1] Edouard Kaminski,[1] Philippe Roux,[3] Yosuke Aoki,[2] Florent Brenguier[3]

September 30, 2019

Supplementary Figures

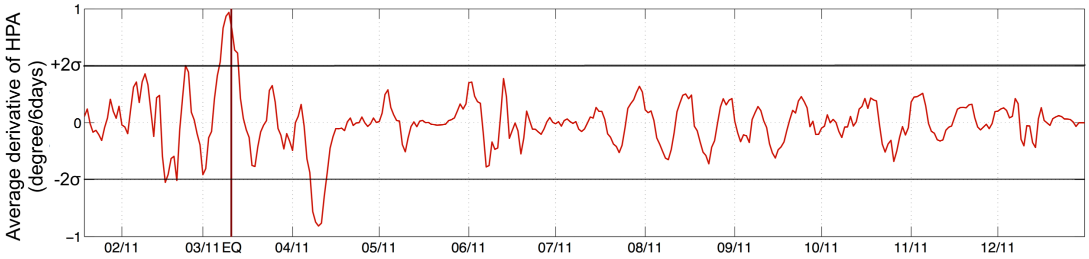

Supplementary Figure 1: Derivative of the horizontal polarization anomaly: First time derivative of the average horizontal polarization anomaly (HPA). Horizontal grey lines, the interval of two standard deviations from the mean of the derivative.

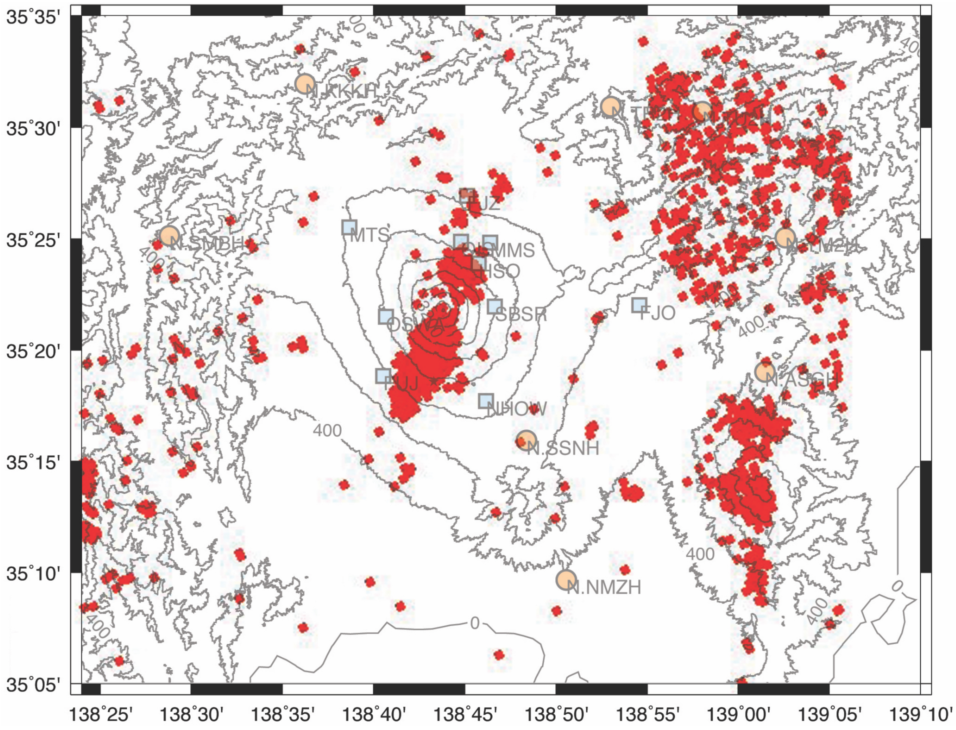

Supplementary Figure 2: Tohoku-Oki earthquake aftershock map: Red points indicate aftershocks that occurred in the study area, with magnitude > 2 and depth < 10 km (Fig. 2b).

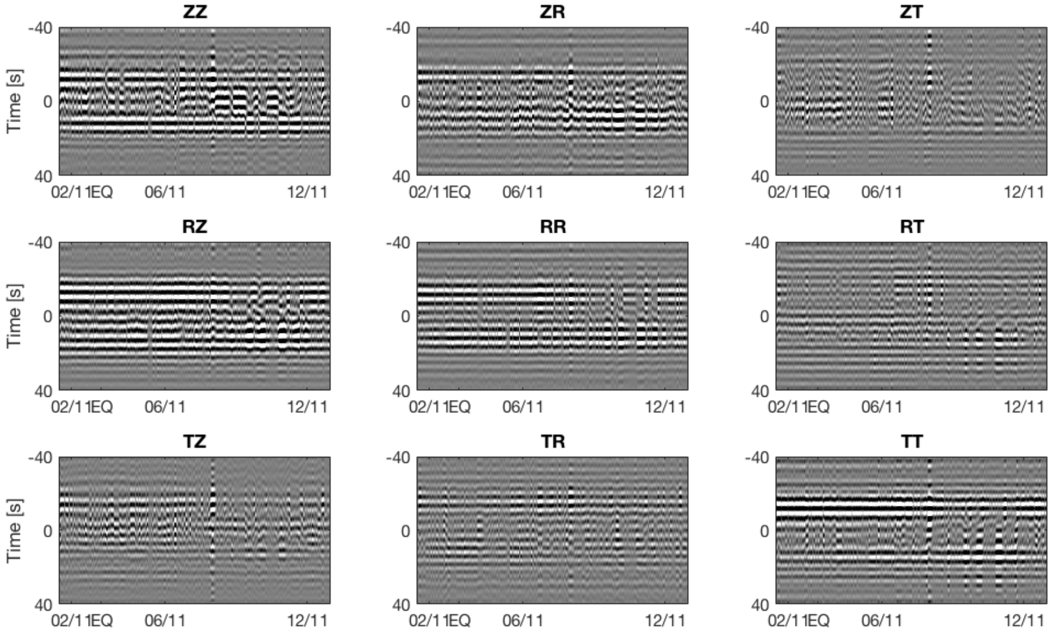

Supplementary Figure 3: Continuous measurements of the cross-correlation tensor: Example of the nine components of the cross-correlation tensors for the SSNH-TR2H receiver pair computed between mid-January and the end of 2011. The asymmetry of the causal and acausal cross-correlations is the result of the non-uniform distribution of the seismic noise. The contributions of the off-diagonal components ZT, RT, TZ, and TR are clearly visible.

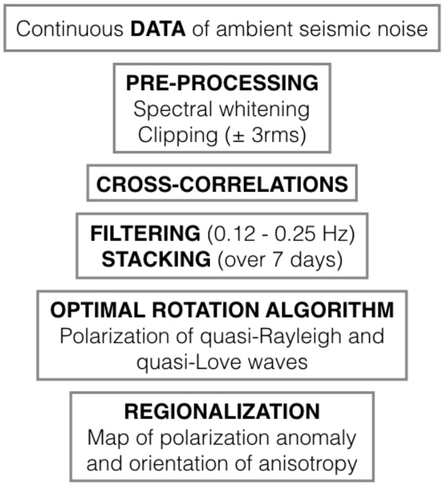

Supplementary Figure 4: Scheme of the data processing followed in this study.

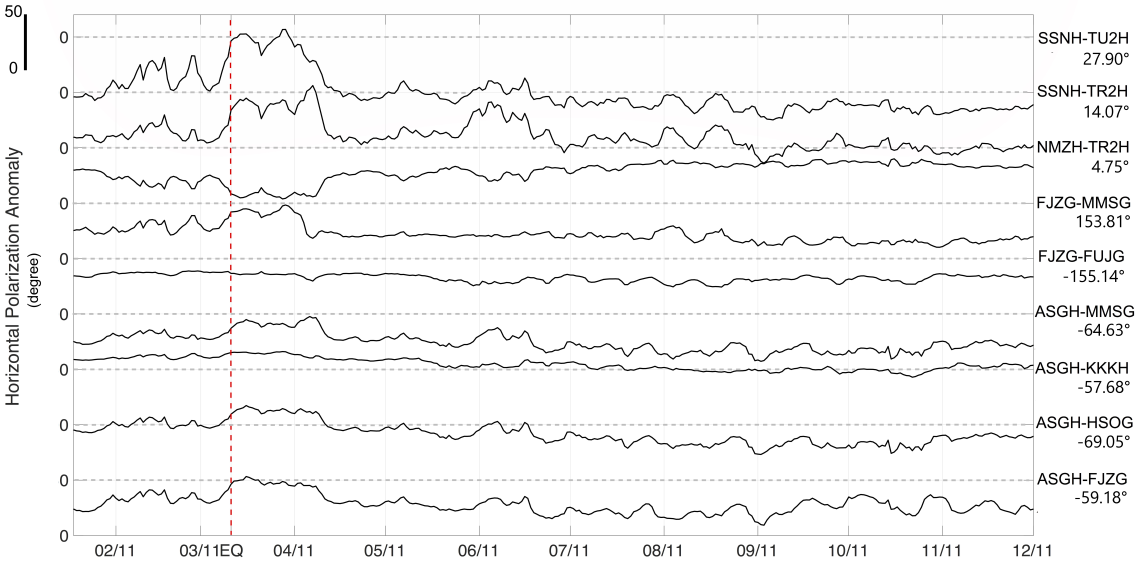

Supplementary Figure 5: Temporal variations of the horizontal polarization anomaly: Continuous measurements of the HPA for some receiver pairs through the year 2011. Vertical red line, time of the Tohoku-Oki earthquake. The numbers below the names of the stations indicate the azimuths of the receiver pairs.

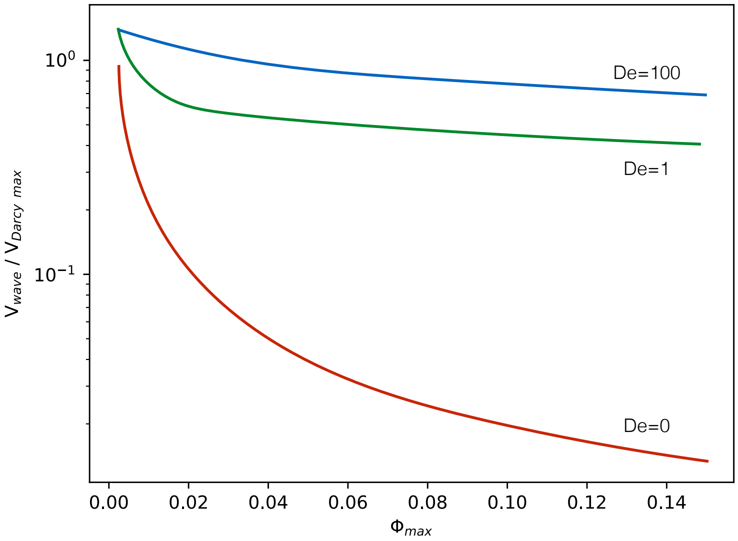

Supplementary Figure 6: Analysis of the speed of a porosity surge: Evolution of the velocity of a porosity surge as a function of the maximum porosity in the wave ϕ_{max} for three values of the Deborah number. The velocity is calculated using Equations (9a) and (9b) of [1]. The velocity is made dimensionless using the Darcy's velocity corresponding to ϕ_{max}.

Supplementary Reference

[1] Vasilyev, O. V., Podladchikov, Y. Y. & Yuen, D. A. Modeling of compaction driven flow in poro-viscoelastic medium using adaptive wavelet collocation method. *Geophysical Research Letters* **25**, 3239–3242 (1998).
